# Calorie Restriction-Induced Increase in Skeletal Muscle Insulin Sensitivity Is Not Prevented by Overexpression of the p55α Subunit of Phosphoinositide 3-Kinase

**DOI:** 10.3389/fphys.2018.00789

**Published:** 2018-06-27

**Authors:** Vitor F. Martins, Shahriar Tahvilian, Ji H. Kang, Kristoffer Svensson, Byron Hetrick, Wallace S. Chick, Simon Schenk, Carrie E. McCurdy

**Affiliations:** ^1^Department of Orthopaedic Surgery, University of California, San Diego, La Jolla, CA, United States; ^2^Biomedical Sciences Graduate Program, University of California, San Diego, La Jolla, CA, United States; ^3^Department of Human Physiology, University of Oregon, Eugene, OR, United States; ^4^Department of Cell and Developmental Biology, University of Colorado Anschutz Medical Campus, Aurora, CO, United States

**Keywords:** Cre-LoxP, 2-deoxyglucose, glucose tolerance, Pik3r1, insulin sensitivity, calorie restriction

## Abstract

**Introduction:** The Phosphoinositide 3-kinase (PI3K) signaling pathway plays an important role in skeletal muscle insulin-stimulated glucose uptake. While whole-body and tissue specific knockout (KO) of individual or combinations of the regulatory subunits of PI3K (p85α, p55α, and p50α or p85β); increase insulin sensitivity, no study has examined whether increasing the expression of the individual regulatory subunits would inhibit insulin action *in vivo*. Therefore, the objective of this study was to determine whether skeletal muscle-specific overexpression of the p55α regulatory subunit of PI3K impairs skeletal muscle insulin sensitivity, or prevents its enhancement by caloric restriction.

**Methods:** We developed a novel “floxed” mouse that, through the Cre-LoxP approach, allows for tamoxifen (TMX)-inducible and skeletal muscle-specific overexpression of the p55α subunit of PI3K (referred to as, ‘p55α-mOX’). Beginning at 10 weeks of age, p55α-mOX mice and their floxed littermates (referred to as wildtype [WT]) either continued with free access to food (*ad libitum;* AL), or were switched to a calorie restricted diet (CR; 60% of AL intake) for 20 days. We measured body composition, whole-body energy expenditure, oral glucose tolerance and *ex vivo* skeletal muscle insulin sensitivity in isolated soleus and extensor digitorum longus muscles using the 2-deoxy-glucose (2DOG) uptake method.

**Results:** p55α mRNA and protein expression was increased ∼2 fold in muscle from p55α-mOX versus WT mice. There were no differences in energy expenditure, total activity, or food intake of AL-fed mice between genotypes. Body weight, fat and lean mass, tissue weights, and fasting glucose and insulin were comparable between p55α-mOX and WT mice on AL, and were decreased equally by CR. Interestingly, overexpression of p55α did not impair oral glucose tolerance or skeletal muscle insulin signaling or sensitivity, nor did it impact the ability of CR to enhance these parameters.

**Conclusion:** Skeletal muscle-specific overexpression of p55α does not impact skeletal muscle insulin action, suggesting that p85α and/or p50α may be more important regulators of skeletal muscle insulin signaling and sensitivity.

## Introduction

Impaired insulin-stimulated glucose disposal is a common metabolic derangement in aged and obese skeletal muscle (Fink et al., 1983; Rowe et al., 1983; Bonadonna et al., 1990; Kohrt et al., 1993; Karakelides et al., 2010), with this insulin resistance being central to the pathophysiology of type 2 diabetes (T2D) (Weyer et al., 1999). At the molecular level, a phosphorylation-based signaling cascade is required for insulin action, with phosphoinositide 3-kinase (PI3K) being central for the propagation of insulin signaling to glucose uptake in skeletal muscle (Lee et al., 1995; Yeh et al., 1995). The class 1A PI3K is a heterodimeric protein that is composed of a catalytic (p110α, β, γ, and δ encoded by *Pik3ca*, *Pik3cb*, *Pik3cg*, and *Pik3cd*, respectively) and a regulatory (p85α, p55α, p50α, encoded by *Pik3r1*; p85β, *Pik3r2*; p55γ, *Pik3r3*) subunit, and is well-known to control many cellular processes including cell growth, proliferation, survival, metabolism, and insulin-stimulated glucose uptake (Fruman et al., 2000; Ueki et al., 2002a; Abell et al., 2005; O’Neill et al., 2007; Pensa et al., 2014a,b). Under normal conditions, the regulatory subunits bind to the catalytic subunit in the cytosol both repressing p110 enzymatic activity and preventing its degradation (Yu et al., 1998; Ueki et al., 2000). Additionally, p85α, but not the other regulatory subunits, forms a homodimer that can stabilize and enhance the activity of the lipid phosphatase, PTEN, further suppressing PI3K activity (Cheung et al., 2015). Several studies (Ueki et al., 2000, 2002a; Brachmann et al., 2005), but not all (Geering et al., 2007), support a model by which the PI3K regulatory subunits are in excess to the catalytic subunits, and therefore, monomeric regulatory subunits can potentially bind to insulin receptor substrate (IRS) proteins, and as a result can compete with PI3K heterodimers for access to IRS proteins under insulin-stimulated conditions. Indeed, certain studies propose that the ratio of catalytic-to-regulatory subunits might modulate the regulation of insulin sensitivity (Ueki et al., 2000, 2002a; Brachmann et al., 2005).

In various models of insulin resistance (high-fat diet [HFD], obesity, diabetes, overexpression of human placental growth hormone [hpGH], and pregnancy), the abundance of p85α, p55α, and p50α is increased in skeletal muscle (Friedman et al., 1999, 2008; Barbour et al., 2004; Bandyopadhyay et al., 2005; McCurdy et al., 2012), adipose tissue (McCurdy et al., 2012), and liver (Kerouz et al., 1997). Furthermore, we have previously demonstrated that caloric restriction enhances skeletal muscle insulin sensitivity in association with a reduction in p50α and p55α (McCurdy et al., 2005; Schenk et al., 2011). Moreover, whole body heterozygous KO of *Pik3r1* (p85α, p55α, and p50α) (Mauvais-Jarvis et al., 2002; McCurdy et al., 2012), p85β KO (Ueki et al., 2002b), or p55α and p50α double KO (Chen et al., 2004) enhances insulin-stimulated Akt phosphorylation and improves insulin action in mice. Importantly, current KO mouse models do not recapitulate the clinical setting in which there is not a ‘loss’ of these proteins, but rather a change in level. Taken together, while these studies suggest an important role for the p85α/p55α/p50α regulatory subunits in regulating insulin action, the precise role of any one subunit on skeletal muscle insulin sensitivity is not yet known; indeed, differences in the domain architecture between p85α, p55α, and p50α suggest non-overlapping functional roles (Ueki et al., 2000; Okkenhaug and Vanhaesebroeck, 2001). To this end, we leveraged Cre/LoxP methodology to develop a novel mouse model that allows tissue-specific, physiological overexpression of the p55α subunit. Accordingly, in this study, we used this model to investigate the role of increased p55α in regulating skeletal muscle insulin action. We hypothesized that overexpression of p55α in skeletal muscle would reduce skeletal muscle insulin sensitivity in chow-fed mice, and prevent improvements in muscle insulin sensitivity induced by a calorie restricted (CR) diet.

## Materials and Methods

### Generation of Floxed p55α Mouse Model

**FIGURE 1:**
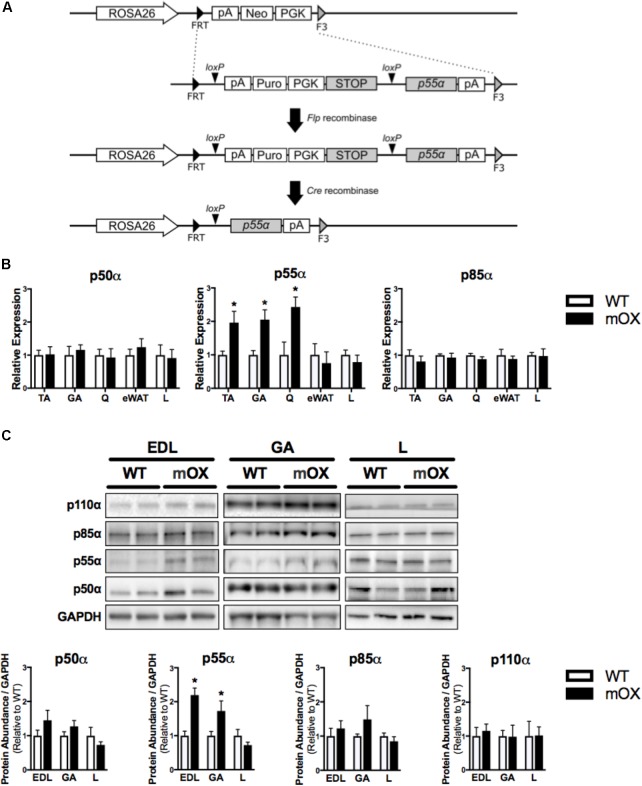
p55α-mOX mice have increased p55α expression in skeletal muscle. **(A)** Strategy for the development of Cre-Lox mediated p55α overexpression mouse model. **(B)** Gene expression of p50α, p55α, and p85α in skeletal muscle (tibialis anterior [TA; *n* = 6/group], gastrocnemius [GA; *n* = 5/group], quadriceps [Q; *n* = 3/group]), epididymal white adipose tissue (eWAT; *n* = 5/group), and liver (L; *n* = 5/group) of male p55α-mOX mice relative to WT mice. **(C)** Quantitation and representative images of total p50α, p55α, p85α, and p110α protein abundance in skeletal muscle (extensor digitorum longus [EDL; *n* = 4/group], GA (*n* = 4/group)) and liver (*n* = 4/group) of male p55α-mOX and WT mice. ^∗^*p* < 0.05 unpaired Student’s *t*-test p55α-mOX versus WT. Data reported as mean ± SEM.

To generate knock-in of *Pik3r1* transcript variant 1 (NM_001024955.2 encodes for the p55α regulatory subunit) at the ROSA26 locus, a recombination cassette was made by flanking the cDNA of *p55*α and a floxed PGK-puromycin selection marker followed by 4x SV40 polyA STOP signal with FRT and F3 (**Figure 1A**). The expression clone for the *Pik3r1* variant (*p55*α) was kindly provided by D.A. Fruman (University of California, Irvine). This cassette together with pCAG-Flpe were electroporated into ROSA-FNF3-1F1 ES cells, an embryonic stem (ES) cell line targeted with FRT-PGK-neo-F3 at the ROSA26 locus. The exchange of neomycin for *p55*α at the ROSA26 locus was facilitated by Flp-recombinase mediated site-specific recombination so that the recombinants would become G418 sensitive and puromycin resistant. The correct exchange was confirmed by PCR. The 4x SV40 polyA STOP signal along with the selection marker PGK-Puro is removed by Cre recombinase placing *p55a* cDNA under the expression of the endogenous ROSA26 promoter. ES cells were injected into C57BL/6J blastocysts. Chimeric mice were crossed to the C57BL/6J line for 3 generations and interbred to generate mice homozygous for the p55 floxed allele. We observed the predicated genetics in offspring suggesting no impairment in viability of mice harboring the targeted alleles. Targeted versus endogenous alleles were identified by PCR with the same forward primer (3′-GCA CTT GCT CTC CCA AAG-5′). The reverse primer, R1, was used to identify targeted alleles (3′-GAC CGA GTA CAA GCC CAC-5′). The reverse primer, R2, was used to identify endogenous alleles (3′-AAA CTC GGG TGA GCA TGT C-5′). Reactions were performed using DreamTaq DNA polymerase (Thermo Fisher EP0702) according to manufacturer’s instructions, except reactions contained 2.5% DMSO final concentration.

### Animals

Studies were conducted in male and female mice on a C57BL/6J background housed in a conventional facility with a 12-h light/12-h dark cycle. Inducible, skeletal muscle-specific, p55α overexpressing mice (p55α-mOX) were generated by crossing mice homozygous for the *p55*α floxed allele (MGI: *6150809*) with mice expressing Cre recombinase (Cre), in a tamoxifen (TMX)-inducible manner, under the human α-skeletal actin (iHSA) promoter (**RRID**: *IMSR_JAX:025750*) (McCarthy et al., 2012). At 8 weeks of age, floxed Cre-negative [i.e., wildtype (WT)] and their floxed, Cre-positive (p55α-mOX) littermates were orally gavaged with TMX (2mg) for five consecutive days. Oral glucose tolerance test (OGTT), whole body respirometry, and *ex vivo* 2-deoxyglucose uptake (2DOGU) assays were performed 4–6 weeks after starting TMX. This study was carried out in accordance with the recommendations of the Institutional Animal Care and Use Committee of UC San Diego. The protocol was approved by the Institutional Animal Care and Use Committee of UC San Diego.

### CR Diet

The CR studies were performed as previously described (Schenk et al., 2011). Briefly, at 9 weeks old, food intake was assessed for 7 consecutive days at 1200 h for individually housed mice given free access to a standard chow diet (Harlan 7912 irradiated chow). At 10 weeks old, mice were randomized to continue AL feeding or were switched to a CR diet (60% of AL) for 20 days. Food was provided daily to CR mice between 1100 and 1200 h.

### qPCR

Total RNA was isolated from skeletal muscle, liver and epididymal white adipose tissue (eWAT) using TRIzol Reagent (Thermo Fisher Scientific, Waltham, MA, United States), Navy Eppendorf RNA lysis kit (Next Advance, Troy, NY, United States), and RNeasy Mini kit (Qiagen, Germantown, MD, United States). RNA concentrations were normalized across samples and the same amount of total RNA used for cDNA synthesis using iScript^TM^ Reverse Transcription Supermix (Bio-Rad, Hercules, CA, United States). Semi-quantitative real-time PCR analysis was performed using iTaq^TM^ SYBR Green master mix (Bio-Rad, Hercules, CA, United States) on a CFX384 Touch^TM^ real-time PCR system (Bio-Rad, Hercules, CA, United States). Relative expression levels for each gene of interest were calculated with the ΔΔC*_t_* method, using eukaryotic elongation factor 2 alpha (*eEF2a)* as the normalization control: 5′-CTG GCA GAG GAC ATC GAT AAG-3′, 5′-GCA ACG TCC CAC TCA TAC TT-3′. Primer sequences used for the different regulatory subunits of PI3K (Abell et al., 2005) were: *p85*α*:* 5′-GCC CCG TGC TTT TCA GAT TTC-3′, 5′-TCC TGC TGG TAT TTG GAC ACT GGG TAG-3′; *p55*α: 5′-GTT ACA GTG CGG GCC GTA TAG GTT TTA-3′, 5′-TCC TGC TGG TAT TTG GAC ACT GGG TAG-3′; *p50*α: 5′-CTG GCA GTT CAA AGC GAA ACC GT-3′, 5′-TCC TGC TGG TAT TTG GAC ACT GGG TAG-3′.

### Immunoblotting

Tissues were homogenized, and immunoblotting was performed after SDS–PAGE, as previously described (LaBarge et al., 2016; Dent et al., 2017; Svensson et al., 2017). The following antibodies were used; Cell Signaling: p110α (CS 4249, **RRID**: *AB_2165248*), Akt (CS 2920, **RRID**: *AB_329827*), phosphorylated (p)Akt^S473^ (CS 4058, **RRID**: *AB_331168*), pAkt^T308^ (CS 9275, **RRID**: *AB_329828*), eEF2a (CS 2332, **RRID**: *AB_2097292*); Millipore Sigma: pan-p85 (ABS 233, **RRID**: *AB_2722790*); Fitzgerald Industries: glyceraldehyde-3-phosphate dehydrogenase (GAPDH; 10R-G109a, **RRID**: *AB_1285808*). Densitometric analysis of immunoblots was performed using Image Lab Software (Bio-Rad, Hercules, CA, United States).

### Energy Expenditure and Body Composition

Whole body energy expenditure was assessed via indirect calorimetry, using the Comprehensive Lab Animals Monitoring System (CLAMS; Columbus Instruments, Columbus, OH, United States). Oxygen consumption (VO_2_), respiratory exchange ratio (RER), total activity, and food intake were continuously measured for 3 consecutive days and values were averaged from the light and dark phases recorded on days 2 and 3. Body composition was assessed by magnetic resonance imaging (MRI; EchoMRI-100^TM^, Houston, TX, United States).

### Blood Glucose and Plasma Insulin Concentrations

After 20 days on their respective diets, blood glucose concentrations were determined from tail vein blood after a 4 h fast using a handheld glucose meter (Ascensia Contour, Bayer HealthCare, Mishawaka, IL, United States). Whole blood was collected with EDTA from the inferior vena cava of anesthetized mice and centrifuged at 5,000 *g* at 4°C for 5 min, and the plasma frozen at -80°C for subsequent determination of plasma insulin using an ELISA kit (80-INSMS-E01; ALPCO Diagnostics, Salem, NH, United States).

### Oral Glucose Tolerance Test (OGTT)

After 14 days on their respective diets, mice were fasted (4 h) and orally gavaged with dextrose (4 g/kg). Blood glucose concentration was measured by tail vein at 0 (before gavage), 20, 30, 45, 60, 90, and 120 min after gavage using a handheld glucose meter (Ascensia Contour, Bayer HealthCare, Mishawaka, IL, United States). Area under the curve (AUC) was calculated using Prism 7 (GraphPad Software Incorporated, La Jolla, CA, United States) using 0 mg/dL as the baseline.

### 2DOG Uptake

Basal and insulin-stimulated (0.36 nM) 2DOG uptake was measured in isolated and paired soleus and extensor digitorum longus (EDL) muscles, as previously described (Schenk et al., 2011; Svensson et al., 2017).

### Statistics

Statistical analyses were performed using Prism 7 (GraphPad Software Incorporated, La Jolla, CA, United States). Data were analyzed using an unpaired Student’s *t*-test or 2-way analysis of variance (ANOVA), with significant differences at *p* < 0.05. Specifically, tissue weights, fasting glucose, fasting insulin, OGTT AUC, 2DOGU, and pAkt were analyzed by two-way ANOVA for the main effects of diet and genotype. For the OGTT, a two-way ANOVA (diet and genotype) was used to compare blood glucose within each time point. For non-diet based comparisons between WT and p55α-mOX mice (e.g., *Pik3r1* mRNA and protein abundance, CLAMS, and MRI), an unpaired Student’s *t*-test was used. All data are expressed as mean ± SEM.

## Results

### Development, Generation, and Validation of the p55α-mOX Mouse Model

p55α mRNA expression was ∼2-fold higher in skeletal muscle (tibialis anterior [TA], gastrocnemius [GA], and quadriceps [Q]) of p55α-mOX versus WT mice, but was comparable in eWAT and liver. p50α and p85α mRNA expression were comparable between p55α-mOX and WT mice across all tissues (**Figure 1B**). Upregulated p55α gene expression was associated with ∼2-fold higher p55α protein abundance in p55α-mOX compared to WT mice (**Figure 1C** and Supplementary Figure 1.2) in the EDL and GA, but was comparable in liver. Similar to the gene expression data, skeletal muscle and liver p50α and p85α protein abundance was not different between p55α-mOX and WT mice (**Figure 1C**). Moreover, the protein abundance of p110α in skeletal muscle and liver was comparable between genotypes (**Figure 1C** and Supplementary Figure 1.1).

### Energy Expenditure, Activity and Food Intake Are Comparable Between p55α-mOX and WT Mice

**FIGURE 2:**
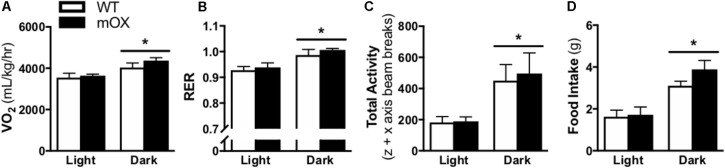
Energy expenditure, activity and food intake is comparable between p55α-mOX and WT mice. **(A–D)** Energy expenditure, spontaneous activity, and food intake measurements were made on male p55α-mOX and WT mice using the CLAMS system over 3 consecutive days and averages for the light and dark cycles on days 2 and 3 are presented. **(A)** Whole body oxygen consumption (VO_2_) and **(B)** Respiratory exchange ratio (RER) were measured by indirect calorimetry. **(C)** Total activity (i.e., total z + x axis beam breaks). **(D)** Cumulative food intake. *n* = 5/group. ^∗^*p* < 0.05 main effect of light cycle. Data reported as mean ± SEM.

Whole-body oxygen consumption (VO_2_), RER, total activity (i.e., total z + x axis beam breaks), and food intake were significantly increased in the dark vs. light phase (main effect, *p* < 0.05), but were not different between genotypes (**Figure 2**).

### Body Mass, Composition, Tissue Weights, and Fasting Glucose and Insulin Are Unaffected by Overexpression of p55α

In AL-fed male (**Table 1**) and female (**Table 2**) mice, there were no differences between genotypes in body, percent fat, percent lean, skeletal muscle (GA and Q), heart, or liver mass, or fasting glucose and fasting insulin. As expected, CR in male mice significantly reduced body mass, fasting glucose and fasting insulin, with no differences noted between WT and p55α-mOX mice (**Table 1**).

**Table 1:** Male mice MRI, tissue weights, and fasting glucose and insulin.

	AL		CR	
		
	WT	mOX	WT	mOX
	*n* = 8	*n* = 6	*n* = 4	*n* = 6
BW (g)	24.7± 0.6	25.6± 0.4	20.6± 1.6*	20.4± 0.8*
Lean Mass (%)	85.2± 1.0	86.5± 0.6	*n.d.*	*n.d.*
Fat Mass (%)	9.9± 1.1	8.4± 0.6	*n.d.*	*n.d.*
GA (mg)	112± 2	118± 4	91± 8*	91± 6*
Q (mg)	171± 3	176± 6	130± 13*	130± 7*
Heart (mg)	112± 4	121± 6	117± 7	102± 4
Liver (mg)	1252± 102	1134± 65	791± 51*	838± 22*
Fasting Glucose (mg/dL)	121± 11	115± 10	77± 10*	80± 2*
Fasting Insulin (ng/mL)	0.74± 0.21	1.67± 0.37	0.51± 0.16*	0.59± 0.27*


*GA, gastrocnemius; Q, quadriceps. n.d., not determined. ^∗^*p* < 0.05 main effect of CR. Data reported as mean ± SEM.*

**Table 2:** Female mice MRI, tissue weights, and fasting glucose and insulin.

	WT	mOX
	*n* = 6	*n* = 5
BW (g)	18.5 ± 0.8	18.7 ± 0.3
Lean Mass (%)	82.9 ± 1.5	82.8 ± 1.8
Fat Mass (%)	12.0 ± 1.3	12.4 ± 1.9
GA (mg)	84 ± 4	82 ± 2
Q (mg)	129 ± 4	131 ± 4
Heart (mg)	113 ± 8	108 ± 4
Liver (mg)	1000 ± 73	1007 ± 35
Fasting Glucose (mg/dL)	129 ± 9	110 ± 6
Fasting Insulin (ng/mL)	0.77 ± 0.27	0.56 ± 0.12


*GA, gastrocnemius; Q, quadriceps. Data reported as mean ± SEM.*

### Glucose Tolerance Is Not Impaired in Male or Female p55α-mOX Mice

**FIGURE 3:**
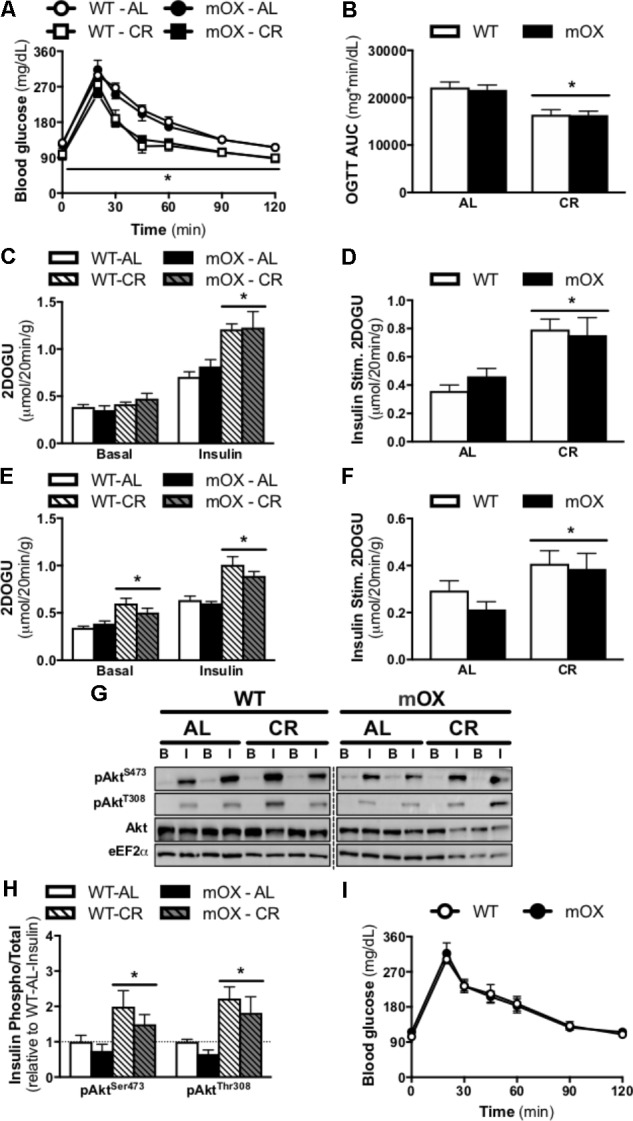
CR-induced increase in glucose tolerance, skeletal muscle insulin sensitivity, and Akt phosphorylation is similar in p55α-mOX and WT mice. p55α-mOX and WT mice were fed a AL or CR diet for 20 days. **(A)** Blood glucose concentrations and **(B)** area under the curve (AUC) of male mice during an oral glucose tolerance test (OGTT; 4 g/kg). WT – AL (*n* = 11), WT – CR (*n* = 4), mOX – AL (*n* = 7), mOX – CR (*n* = 6). **(C–F)** Insulin-stimulated (0.36 nmol/L) 2-deoxy-glucose uptake (2DOGU) in isolated soleus and EDL muscles from male mice. WT – AL (*n* = 8), WT – CR (*n* = 4), mOX – AL (*n* = 6), mOX – CR (*n* = 6). Basal and insulin 2DOGU in **(C)** soleus and **(E)** EDL muscles. Insulin-stimulated (Insulin Stim.) 2DOGU (calculated as insulin 2DOGU – basal 2DOGU) in **(D)** soleus and **(F)** EDL muscles. **(G)** Phospho-Akt^S473^ (pAkt^S473^), phospho-Akt^T308^ (pAkt^T308^), total Akt, and eEF2α in basal and insulin-stimulated (B and I, respectively) EDL muscles. The dashed line represents a single lane with protein ladder which was omitted. **(H)** Quantification of pAkt^S473^ and pAkt^T308^ compared to total protein abundance of Akt in the EDL muscle. WT – AL (*n* = 6), WT – CR (*n* = 4), mOX – AL (*n* = 6), mOX – CR (*n* = 6). **(I)** Blood glucose concentrations of female mice during an OGTT (4 g/kg). WT (*n* = 9), mOX (*n* = 6). Data reported as mean ± SEM. ^∗^*p* < 0.05 main effect of CR (within a time point for OGTT).

Blood glucose concentrations and the AUC during an OGTT were comparable between AL-fed male (**Figures 3A,B**) and female mice [**Figure 3I**; AUC: (21,601 ± 1,215 vs. 21,511 ± 1,543, WT vs. p55α-mOX, *P* > 0.05)] regardless of genotype. In male mice, CR significantly improved glucose tolerance, and this improvement was comparable between p55α-mOX and WT mice.

### Overexpression of p55α Does Not Affect Skeletal Muscle Insulin Sensitivity

In AL-fed male mice, 2DOG uptake in the presence of insulin and insulin-stimulated 2DOGU (i.e., insulin 2DOGU – basal 2DOGU) was not impacted by p55α overexpression in either the soleus (**Figures 3C,D**) or EDL (**Figures 3E,F**). As expected, CR enhanced insulin sensitivity in the soleus (**Figures 3C,D**) and EDL (**Figures 3E,F**), with this enhancement comparable between genotypes. Notably, basal 2DOG uptake was comparable in AL-fed mice in both muscles, however, it was increased by CR, in both WT and p55α-mOX mice in the EDL (**Figure 3E**), but not the soleus (**Figure 3C**). In line with the insulin-stimulated 2DOGU data in AL- and CR-fed mice, insulin-stimulated phosphorylation of Akt (S473 and T308) was comparable between p55α-mOX and WT mice, with values being ∼2-fold higher in CR mice (**Figures 3G,H** and Supplementary Figure 2). The dashed line represents a single lane with protein ladder which was omitted.

## Discussion

The stoichiometry between the catalytic and regulatory subunits of PI3K modulate insulin signaling via the competition for binding to IRS proteins between inactive monomeric regulatory subunits and active heterodimeric PI3K (Ueki et al., 2000, 2002a; Brachmann et al., 2005). Although many studies have investigated the metabolic effects of global and tissue-specific deletion of regulatory subunit(s) of PI3K, to date, none have assessed the direct role of any one subunit on skeletal muscle insulin sensitivity. Herein, we developed a novel mouse model that allows tissue-specific overexpression of the p55α regulatory subunit of PI3K, and sought to study its role in modulating skeletal muscle insulin sensitivity. Interestingly, our results demonstrate that a 2-fold increase in p55α in skeletal muscle does not alter insulin sensitivity in AL-fed mice, nor does it prevent the CR-induced improvements in skeletal muscle insulin sensitivity.

To directly determine the role of p55α in skeletal muscle biology, we generated and characterized mice with muscle-specific, and inducible, OX of the p55α subunit of PI3K. Our model is novel as it is the first *in vivo* assessment of increased expression of any PI3K regulatory subunit. This provides a unique advantage, compared to others, as increasing the abundance of one subunit is perhaps more physiologically relevant than removing one or multiple subunits completely. Furthermore, being an inducible model allows us to circumvent any potentially confounding effects of PI3K on development (Bi et al., 1999; Fruman et al., 2000; Yoshioka et al., 2012). This model is further advantageous as, thus far, p55α expression has only been deleted in conjunction with deletion of the other regulatory subunits (Fruman et al., 2000; Mauvais-Jarvis et al., 2002; Chen et al., 2004), making it difficult to probe the differential functional roles of the regulatory subunits. To this end, in p55α-mOX mice, there was only upregulation of the mRNA and protein expression of p55α, with no effect seen on p50α, p85α, or p110α. Indeed, the ∼2-fold higher abundance of p55α in muscle from p55α-mOX as compared to WT is comparable to differences in p55α abundance when comparing muscle from lean, insulin sensitive and obese, insulin resistant individuals (Bandyopadhyay et al., 2005). Thus, our mouse model induces a physiologically relevant increase in p55α abundance and, as such, is appropriate for discerning the contribution of p55α to skeletal muscle insulin action.

While PI3K is required for insulin-stimulated glucose uptake in skeletal muscle (Lee et al., 1995; Yeh et al., 1995), the contribution of each regulatory subunit to this process has not been fully defined. Whole-body reductions in PI3K regulatory subunit abundance in various transgenic mouse models [heterozygous KO of *Pik3r1* (McCurdy et al., 2012), p85β KO (Ueki et al., 2002b), or p55α and p50α double KO (Chen et al., 2004)] have been associated with enhanced insulin-stimulated Akt phosphorylation and insulin action in skeletal muscle (Vanhaesebroeck et al., 2005). When considering tissue-specific models, however, there are diverse responses to reducing PI3K regulatory subunit abundance. For example, mice with liver-specific KO of *Pik3r1* demonstrate improved glucose tolerance and insulin-stimulated glucose uptake in skeletal muscle (Taniguchi et al., 2006) and are protected against HFD-induced glucose intolerance (Taniguchi et al., 2007). Together these data suggest that p85β in liver is able to compensate for the loss of all *Pik3r1* subunits and perhaps is more efficient at insulin-mediated signaling. In contrast to the liver, mice with a skeletal muscle-specific KO of *Pik3r1* exhibit no change in insulin-stimulated Akt phosphorylation but a decrease in insulin-stimulated PI3K activity and reduced insulin-stimulated 2DOG uptake in the EDL after supraphysiological insulin stimulation (50 mU/mL), but not a sub-maximal (0.6 mU/mL insulin) concentration; insulin sensitivity in the soleus is unaffected with either insulin concentration (Luo et al., 2006). Correspondingly, states of insulin resistance are associated with increases in PI3K regulatory subunit abundance. Obese and type 2 diabetic humans have ∼2-3-fold higher abundance of all *Pik3r1* protein isoforms in skeletal muscle, as compared to lean, non-diabetics (Bandyopadhyay et al., 2005). Similarly, pregnancy in humans (Friedman et al., 1999, 2008), overexpression of hpGH in mice (Barbour et al., 2004) so as to mimic whole-body insulin resistance during pregnancy, and HFD in mice (McCurdy et al., 2012) increases p85α protein abundance in skeletal muscle. Considering the inverse correlation between PI3K regulatory subunit abundance and skeletal muscle insulin sensitivity, we hypothesized that the overexpression of p55α in skeletal muscle would cause skeletal muscle insulin resistance. However, in the present study, we observed no differences in fasting blood glucose, fasting plasma insulin, whole body glucose tolerance, or insulin-stimulated 2DOG uptake or Akt phosphorylation in soleus or EDL muscles in p55α-mOX versus WT mice. Lastly, in different rodent models of insulin resistance and PI3K subunit modulation, male and female mice can exhibit distinct phenotypes (Clark et al., 1983; Macotela et al., 2009; Saito et al., 2016). Similar to male mice, however, oral glucose tolerance was not affected in female p55α-mOX versus WT mice. Thus, OX of p55α alone in skeletal muscle is not sufficient to mimic an insulin resistant phenotype in mice. The *Pik3r1* regulatory subunits (p85α, p55α, and p50α) have distinct domain architecture suggesting potentially different functional roles (Ueki et al., 2000; Okkenhaug and Vanhaesebroeck, 2001). Hence, a possible reason for observing no difference in insulin sensitivity in the p55α-mOX mouse could be that p85α and/or p50α are more important regulators of skeletal muscle insulin signaling and sensitivity.

CR robustly improves glucose tolerance and enhances skeletal muscle insulin signaling and sensitivity (McCurdy and Cartee, 2005; McCurdy et al., 2005; Schenk et al., 2011; Sharma et al., 2011). We have previously demonstrated that enhanced skeletal muscle insulin signaling (at the level of PI3K-Akt) and sensitivity in mouse and rat models of CR occurs in parallel with a ∼30–40% decrease in p55α and p50α (but not p85α) protein abundance (McCurdy et al., 2005; Schenk et al., 2011). Thus, we hypothesized that OX of p55α in skeletal muscle would, at the very least in part, impair CR-induced improvements in glucose tolerance and skeletal muscle insulin sensitivity. Contrasting this hypothesis, CR potently and equally improved fasting blood glucose, fasting plasma insulin, glucose tolerance, and insulin-stimulated Akt phosphorylation and glucose uptake in skeletal muscle of WT and p55α-mOX mice. Thus, physiologically relevant overexpression of p55α alone in skeletal muscle is not sufficient to diminish the beneficial effects of CR on insulin action, with perhaps overexpression of both p55α and p50α being needed to mitigate these effects.

Modulation of PI3K regulatory and catalytic subunit abundance can affect whole-body energy expenditure, body weight and body composition (Chen et al., 2004; Xu et al., 2010; Becattini et al., 2011; Nelson et al., 2014; Saito et al., 2016). For example, mice with KO of p110α in steroidogenic factor-1 neurons of the ventromedial hypothalamic nucleus (Xu et al., 2010; Saito et al., 2016) or white and brown adipose tissue (Nelson et al., 2014) exhibit decreased energy expenditure, and increased body weight and body fat percentage. Conversely, whole body p55α/p50α double KO mice exhibit decreased body fat percentage, but no change in body weight (Chen et al., 2004). In contrast, however, whole body *Pik3r1* heterozygous KO, and p85β KO mice demonstrate no changes in body weight or percent body fat (McCurdy et al., 2012). Lastly, modulation of other classes of PI3K (e.g., class IB [PI3Kγ]), can also alter energy expenditure (Becattini et al., 2011). In the present study, however, we observed no differences in body, fat, or lean mass, energy expenditure, or activity in p55α-mOX versus WT mice. Thus, although modulation of PI3K both centrally and/or peripherally can alter whole-body energy metabolism and body composition, increasing p55α abundance in skeletal muscle is without effect.

## Conclusion

We developed a new mouse model to investigate the contribution of the p55α regulatory subunit of PI3K to skeletal muscle insulin action. Our results demonstrate that OX of p55α in skeletal muscle does not impact skeletal muscle insulin signaling or sensitivity in AL- or CR-fed mice. However, future studies may reveal a phenotype under other conditions that alter insulin sensitivity such as HFD or post-exercise. Furthermore, future studies that combine knockout and knockin transgenic models to dissect the separate and/or combined effects of modulating the expression of the various regulatory subunits in key metabolic tissues will be of great value given the essential role of PI3K in many cellular processes and the limited knowledge of its regulation.

## Author Contributions

CM, SS, and VM were responsible for the conception and design of the study and the analysis and interpretation of the data. VM was responsible for the design and drafting of the manuscript. CM and SS revised the manuscript critically. WC designed the targeting constructs and produced the mouse embryonic stem cells. ST, JK, KS, and BH contributed to analysis, interpretation of data, and critical revision of the manuscript. All authors gave final approval.

## Conflict of Interest Statement

The authors declare that the research was conducted in the absence of any commercial or financial relationships that could be construed as a potential conflict of interest. The handling Editor declared a past co-authorship with several of the authors BH, SS, and CM.
