# Supplementary material for: Calorie Restriction-Induced Increase in Skeletal Muscle Insulin Sensitivity Is Not Prevented by Overexpression of the p55α Subunit of Phosphoinositide 3-Kinase
Source: Front Physiol. 2018 Jun 27;9:789. doi: 10.3389/fphys.2018.00789 (PMC6030672; doi:10.3389/fphys.2018.00789)
Supplement: Supplementary file 1 [file Data_Sheet_1.DOCX]

Supplementary Material

**Calorie Restriction-Induced Increase in Skeletal Muscle Insulin Sensitivity is not Prevented by Overexpression of the p55α Subunit of Phosphoinositide 3-Kinase**

**Vitor F. Martins^1,2^, Shahriar Tahvilian^1^, Ji H. Kang^1^, Kristoffer Svensson^1^, Byron Hetrick^3^, Wallace S. Chick^4^, Simon Schenk^1,2,*^, Carrie E. McCurdy^3,*^**

***Correspondence:**

C.E. McCurdy, Department of Human Physiology, University of Oregon, 1240 University of Oregon, 122c Esslinger Hall, Eugene, OR 97403, USA

Email: cmccurd5@uoregon.edu

S. Schenk, Department of Orthopaedic Surgery, School of Medicine, University of California San Diego, 9500 Gilman Drive MC0863, La Jolla, CA 92093, USA.

E-mail: sschenk@ucsd.edu

1. **Original gels of expression of catalytic and regulatory subunits of PI3K represented in Figure 1C.**


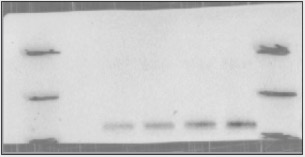

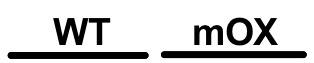


100 kD

150 kD

250 kD

**Supplementary Figure 1.1** Expression of p110α in EDL muscles.

**Supplementary Figure 1.2** Expression of p85α/p55α/p50α in EDL muscles.

**2. Original gels of insulin signaling proteins represented in Figure 3G.**

50 kD

37 kD


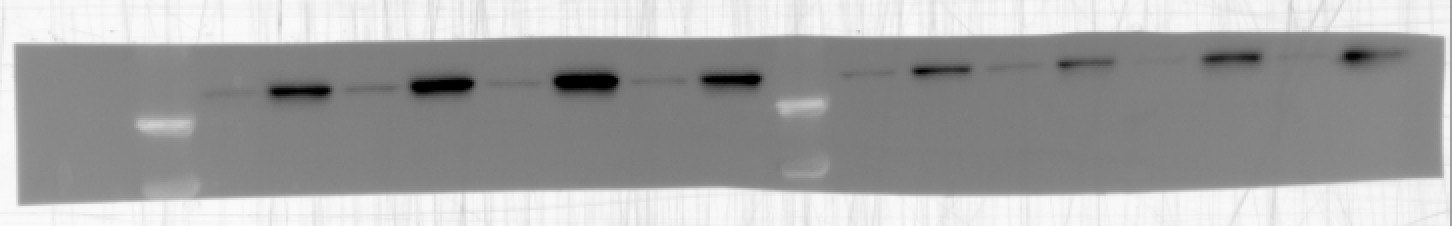


**Supplementary Figure 2.1** Expression of pAkt (S473) in EDL muscles.

50 kD

37 kD


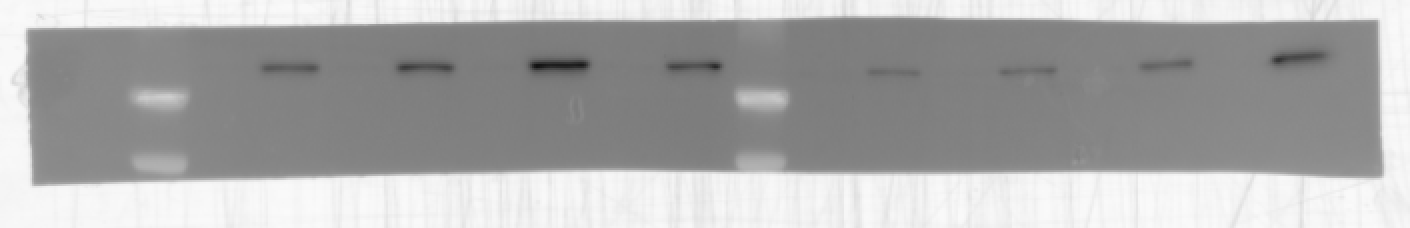


**Supplementary Figure 2.2** Expression of pAkt (T308) in EDL muscles.

50 kD

37 kD


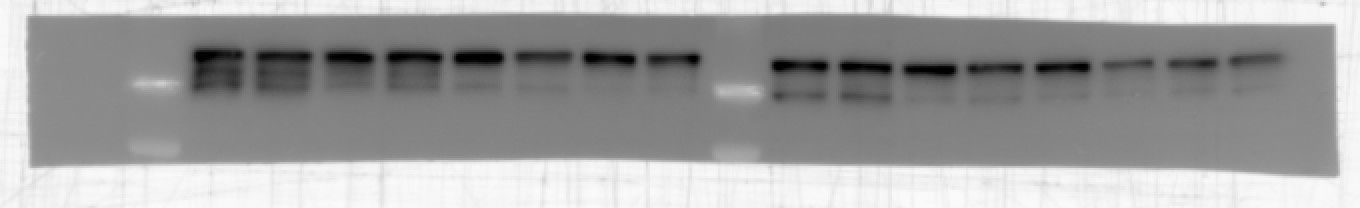


**Supplementary Figure 2.3** Expression of Akt in EDL muscles.


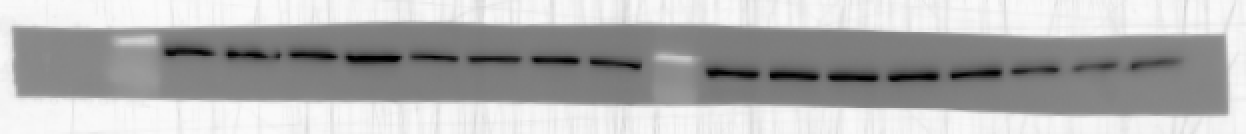


100 kD

75 kD

**Supplementary Figure 2.4** Expression of eEF2 in EDL muscles.
